# Causal links between gut microbiota, plasma metabolites, and insomnia: Insights from Mendelian randomization

**DOI:** 10.1080/19585969.2026.2636470

**Published:** 2026-02-27

**Authors:** XuWen Zheng, Jin Xu, JinNan Yin, JinNuo Fan, Yan Gong, JianMin Yang

**Affiliations:** Department of Emergency, Wujin Hospital Affiliated with Jiangsu University and Wujin Clinical College of Xuzhou Medical University, Changzhou, Jiangsu, China

**Keywords:** Gut microbiota, insomnia, Mendelian randomisation

## Abstract

**Objectives:**

This study explored the plasma metabolites’ mediation effect between gut microbiomes and insomnia through Mendelian randomisation (MR).

**Methods:**

Using publicly accessible GWAS data from 5959 individuals for gut microbiota and 8299 individuals for plasma metabolites, we employed MR analysis to explore their causal effects on insomnia. Insomnia outcome data were obtained from Pan-UKB, GERA, and FinnGen, covering 9007 cases and 871,802 controls. Mediation effects of identified bacterial taxa on insomnia through plasma metabolites were computed using the product of coefficients approach.

**Results:**

Our MR analysis included participants with a mean age of 45.7 years (SD = 11.5) for gut microbiota and 63 years (range 45–85) for plasma metabolites. The analysis identified 10 gut microbiomes and 35 plasma metabolites potentially associated with insomnia respectively. Specifically, increased abundances of certain gut microbiomes, such as species *CAG-145 sp000435615*, were linked to a higher risk of insomnia. Mediation analysis revealed that the plasma metabolite 3-ethylcatechol sulphate levels significantly mediated the effects of these microbiomes on insomnia, explaining up to 31.49% of the total effect.

**Conclusion:**

This study highlights the role of gut microbiota in influencing insomnia risk, mediated through specific plasma metabolites. These findings provide valuable insights into the gut-brain axis and may inform the development of therapeutic targets for managing sleep disorders.

## Introduction

Insomnia affects a substantial portion of the global population, with variations in prevalence based on demographic factors (Lucena et al. 2020). In addition to presenting with challenges in starting and sustaining sleep, early morning awakenings, and sleep that does not serve to restore energy, individuals with insomnia often have excessive daytime sleepiness, fatigue, irritability, and cognitive impairments (Dean et al. 2023). These symptoms severely impact daily functioning and quality of life, contributing to decreased productivity and increased accident risks (Riemann et al. 2022). The aetiology of insomnia is complex, including genetic, psychological, and environmental contributing elements. Neurobiological models indicate that hyperarousal, marked by increased activity in the hypothalamic-pituitary-adrenal (HPA) axis, is essential (Van Someren 2021). Psychological stress and maladaptive behaviours, such as poor sleep hygiene, further exacerbate the condition (Riemann et al. 2022).

The association between gastrointestinal microbiomes and insomnia has become a prominent area to be studied nowadays. Recent studies emphasise the reciprocal association between gut microbiota and insomnia. The gut-brain axis, a communication network between the gastrointestinal tract and the central nervous system, is pivotal to this connection. Alterations in gut microbiota may affect sleep patterns, while sleep disruptions can modify the makeup of gut microbiota (Nie and Tian 2022). Several observational studies have explored the diversity of gut microbiota in individuals with insomnia. For example, research involving 16S rRNA sequencing has identified significant differences in the composition of gut microbiota between insomnia patients and healthy controls (Masyutina et al. 2021). Mendelian randomisation (MR) studies also have provided insights into the causal relationships between gut microbiota and insomnia (Yue et al. 2023). The gut microbiota exerts its effects on the host through various metabolites, but the specific metabolites influencing insomnia remain unclear.

To address this gap in knowledge, we plan to perform a mediation MR analysis, which is a statistical technique used in epidemiology and genetics that employs genetic variants as instrumental variables (IVs) to assess the causal effect of a modifiable exposure on an outcome. The core principle of MR is that genetic variations, randomly assigned at conception, can be utilised to infer causality between exposures and outcomes. This approach minimises confounding factors and reverse causation, provided specific assumptions are met (Burgess and Thompson 2015). We aim to determine whether specific plasma metabolites mediate the relationship between the gastrointestinal microbiomes and insomnia using MR. By examining the effects of genetic variations on both the gastrointestinal microbiota and plasma metabolites, we hope to gain a more comprehensive understanding of their roles in insomnia. This insight has the potential to revolutionise the management of insomnia by facilitating the generation of innovative diagnostic and treatment approaches.

## Materials and methods

### Study design

**Figure 1.**
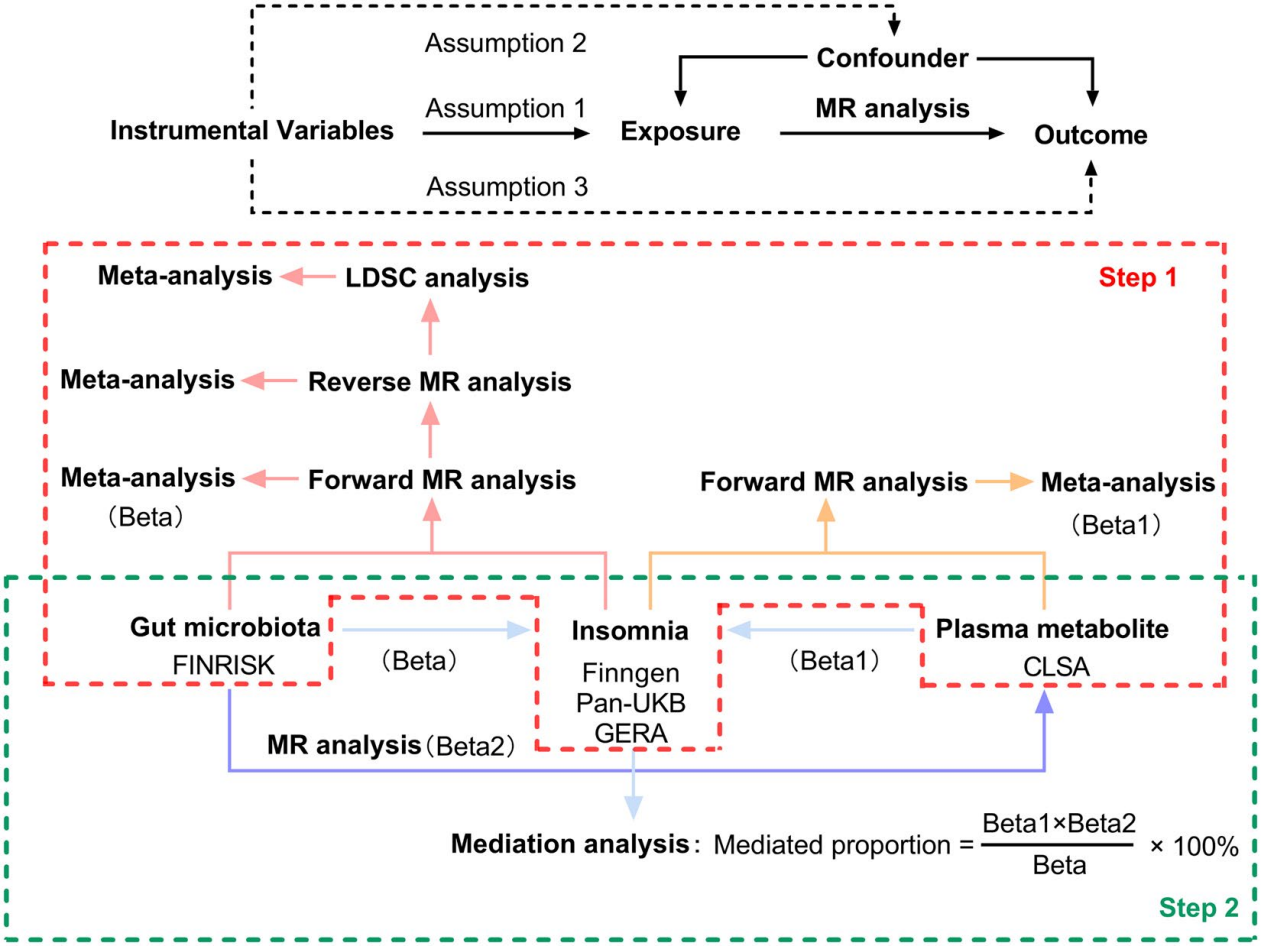
Three assumptions of MR analysis and overview of the study design. The study involves two main steps: Step 1 involves meta-analyses of various datasets, such as FINRISK, FinnGen, Pan-UKB, GERA, and CLSA, along with LDSC, reverse MR, and forward MR analyses to establish associations between gut microbiota, insomnia, plasma metabolites, and their outcomes. Step 2 includes mediation analysis to assess the mediated proportion of the exposure’s effect on the outcome through intermediates like plasma metabolites. This comprehensive MR framework aims to clarify the causal relationships between exposures (gut microbiota, insomnia) and outcomes (health conditions) and explore the role of plasma metabolites in these pathways. MR: Mendelian randomisation; LDSC: Linkage Disequilibrium Score Regression.

All summary-level data used in our analysis are publicly accessible. The design of this study is illustrated in Figure 1. Initially, we explored the relationship between gastrointestinal microbiomes and insomnia using forward and reverse MR, and Linkage Disequilibrium Score Regression (LDSC) analyses. Concurrently, we assessed plasma metabolites associated with insomnia through forward MR analyses. We integrated the results from these MR and LDSC analyses in a meta-analysis to comprehensively evaluate insomnia using diverse data sources. In the second phase, we compared the gastrointestinal microbiomes identified previously with the associated plasma metabolites. We applied the product of coefficients method to assess the mediation effects (ME) of these microbiomes on insomnia mediated by plasma metabolites (VanderWeele 2016). Our analytical procedures adhered to the STROBE-MR guidelines (Skrivankova et al. 2021). Ethical approval was secured for all included studies, and consent was obtained from all participants, in compliance with the guidelines of their institutional review boards or ethical committees respectively.

Gut microbiota data were obtained from the FINRISK study, involving 5,959 participants of European descent. Stool samples were collected under standardised conditions, including detailed instructions provided to participants regarding diet, medication use, and avoidance of antibiotics within a specific timeframe before sample collection. Participants collected faecal samples at home, immediately froze them, and delivered the samples under controlled conditions to preserve microbial composition. Gut microbiome profiles were generated through whole-genome shotgun metagenomic sequencing, resulting in the identification of 473 unique bacterial taxa (Qin et al. 2022). Plasma metabolite data were derived from the Canadian Longitudinal Study on Ageing (CLSA), which involved 8299 participants of European descent. Plasma samples were collected after an overnight fast to ensure consistency in metabolic profiles, with participants instructed to fast for at least 8 h prior to sample collection. Plasma metabolites were quantified using high-throughput techniques, including liquid chromatography-mass spectrometry (LC-MS) and nuclear magnetic resonance (NMR) spectroscopy (Chen et al. 2023). Information related to insomnia was compiled from several key sources, including the FinnGen GWAS Release 10 (Kurki et al. 2023), the Genetic Epidemiology Research on Ageing (GERA) cohort (Guindo-Martínez et al. 2021), and the Pan-UKB GWAS Version 0.4 (Karczewski et al. 2024). Insomnia cases were identified using ICD-10 codes (F51.0, G47.0) for insomnia and related sleep disorders, with a total of 9007 insomnia cases and 871,802 controls. Participants in these datasets were assessed for insomnia symptoms, such as difficulty falling asleep, waking up frequently during the night, and early morning awakenings, which are characteristic of insomnia. In addition to insomnia, data on other sleep disorders were also included, though these were not the focus of the current study. Information on sleep duration was not specifically provided for the insomnia cases, but it is generally considered as part of the diagnostic criteria in the included datasets. As this study focused on the causal relationship between gut microbiota and insomnia, sleep quality and other sleep disorders were not further detailed in our analysis. Detailed diagnostic criteria, adjustments, and sample sizes are presented in Table 1. An online tool was used to estimate the power of the MR analysis (Brion et al. 2013). Given that our sample data were sourced from five distinct databases, we effectively mitigated the risk of sample overlap.

**Table 1.** Detailed information on used summary-level data.

Exposure or outcome	Unit	Consortium	Participants included in analysis	Age (year)	Male (%)	Adjustments	ICD	PMID	Web source
Gut microbiota	SD	FINRISK	5959 European individuals	45.7 ± 11.5	50	age, sex, genotyping batch and top ten genetic principal components		35115689	https://www.ebi.ac.uk/gwas/
Plasma metabolite	SD	CLSA	8299 European individuals	63 (45–85)	52	age, sex, hour since last meal or drink, genotyping batch and the first ten genetic principal components		36635386	https://www.ebi.ac.uk/gwas/
Insomnia	One-unit in log-transformed odds ratio of insomnia	FinnGen	4801 cases and 405,229 controls of European ancestry	53 ± 18	44	sex, age, genotyping batch and ten principal components	ICD-10: F51.0, G47.0	36653562	https://r10.finngen.fi/
		Pan-UKB	234 cases and 413,908 controls of European ancestry	55.1 ± 7.6	48	sex, age, genotyping array, and the first 8 principal components	Phecode: 327.4		https://pan.ukbb.broadinstitute.org/downloads/
		GERA	3972 cases and 52,665 controls of European ancestry	63 (19 to over 100)	42	seven derived principal components, sex, and age	ICD-9: 307.41, 307.42, 327.0, 327.00, 327.01, 327.02, 327.09, 780.52	33893285	http://cg.bsc.es/gera_summary_stats/

To conduct MR analysis, IVs must meet three essential criteria: (1) The selected genetic variants should be robustly linked to the exposure being studied; (2) These variants must be independent of other factors that could influence the outcome to avoid confounding; and (3) The relationship between the genetic variants and the outcome should occur exclusively through the exposure in question (Burgess and Thompson 2015). When using a threshold of *p* < 5 × 10^−8^ and applying a stringent linkage disequilibrium (LD) clumping setting with a 10,000 kb distance and *r*^2^ < 0.001 between IVs, we found that only 284 bacterial taxa and 839 plasma metabolites had total of 324 and 2,230 single nucleotide polymorphisms (SNPs) meeting these criteria respectively. To satisfy the first MR analysis assumption, the exposure identified by GWAS was subjected to a threshold of *p* < 5 × 10^−6^ (Sanna et al. 2019). For each SNP, the F-statistic was calculated using the formula F = beta^2^/se^2^, ensuring that weak IVs had a reduced influence. SNPs with an F-statistic lower than 10 were excluded from the analysis (Bowden et al. 2019; Xie et al. 2023). To ensure consistency, the effect alleles were aligned between the exposure and outcome datasets, excluding any mismatched alleles. Furthermore, ambiguous palindromic SNPs with a minor allele frequency (MAF) close to 0.5 were also discarded to avoid errors. Proxy SNPs were not employed to substitute for missing IVs, as a small percentage of instruments were absent and had minimal impact on the results. In order to detect pleiotropy, we implemented MR pleiotropy residual sum and outlier (MR-PRESSO) test and MR-Egger intercept test. We excluded MR estimates that exhibited significant horizontal pleiotropy from the meta-analysis. Lastly, in order to maintain the third MR postulate, SNPs that were significantly linked to outcome (*p* < 5 × 10^−6^) were excluded. After the initial IVs selection, the forward MR analyses included 1178 plasma metabolites and 416 bacterial taxa. Supplementary Tables S1 and S2 contains a comprehensive inventory of IVs that are associated with all bacterial taxa and plasma metabolites.

### Statistical analysis

Under the random-effects framework, the main MR estimates were calculated using the Inverse-Variance Weighted (IVW) method. When all genetic variants meet the fundamental MR assumptions, the most precise estimates can be obtained because the IVW method is most efficient in the absence of horizontal pleiotropy (Burgess and Thompson 2015). Additionally, three sensitivity analyses were conducted: the weighted median, MR-Egger, and MR-PRESSO. When directional pleiotropy is suspected, MR-Egger is highly valuable, as it can test for pleiotropy through the intercept term and provide more conservative estimates. A significant intercept value indicates the presence of directional pleiotropy (Burgess and Thompson 2017). The weighted median method is less susceptible to invalidity than the IVW method and provides more reliable estimates, especially suitable when minor pleiotropy exists (Bowden et al. 2016). If pleiotropy is present or suspected, MR-PRESSO enhances the accuracy of causal estimations by excluding the impact of outlier variations that violate the assumption of exclusion limitation (Verbanck et al. 2018). Cochran’s Q test was applied to assess heterogeneity among the SNPs. To further investigate horizontal pleiotropy, the MR-Egger intercept test was performed. SNPs with a P-value of less than 0.05 in either the intercept or global test, indicating significant pleiotropy, as well as cases involving fewer than four SNPs, were omitted from the meta-analysis because MR-PRESSO requires a minimum of four SNPs. Finally, the estimates from the IVW method and sensitivity analyses were synthesised using a fixed-effects meta-analysis. However, IVW results showing significant heterogeneity or inconsistencies with sensitivity analyses were excluded. We used LDSC to study the genetic correlation between insomnia and previously identified gut microbiota. Using HapMap3 as reference, non-SNP and SNPs with a MAF below 0.01, duplications, and SNPs with ambiguous strand orientation were excluded from the GWAS data. LDSC effectively identifies genetic correlations by analysing the links between LD and test statistics (Bulik-Sullivan et al. 2015). Genetic correlation is determined by multiplying the z-values of the variants for one trait with the z-values for another trait and then regressing these products on LD scores (Wielscher et al. 2021).

Mediation analysis was conducted to investigate whether plasma metabolites mediate the relationship between gut microbiota and insomnia. The analysis followed a two-step approach, applying the product of coefficients method, a widely used technique for estimating ME in MR studies (Carter et al. 2021). In the first step, we assessed the direct relationship between gut microbiota and insomnia, and between plasma metabolites and insomnia, using forward MR analysis. In the second step, we examined the mediation effect of plasma metabolites in the relationship between identified gut microbiota and insomnia (VanderWeele 2016). The ME was calculated as the product of the coefficients of the microbiome-metabolite and metabolite-insomnia associations (Carter et al. 2019). The proportion of the total effect mediated by metabolites was estimated by dividing the mediation effect by the total effect of gut microbiota on insomnia. The significance of the mediation effect was determined using bootstrap confidence intervals (CI), and a *p* value of < 0.05 was considered statistically significant. Mediation analysis was conducted for the specific pairs of gut microbiomes and plasma metabolites that were identified in the forward MR analysis.

To control the false discovery rate, Bonferroni correction was applied in the meta-analyses of MR studies (Curtin and Schulz 1998). For gut microbiota and plasma metabolites respectively, causal associations were considered significant when IVW p-values were less than 1.20 × 10^−4^ (0.05/416) and 4.24 × 10^−5^ (0.05/1178), and were considered suggestive if IVW *p* values were between the abovementioned thresholds and 0.05. The TwoSampleMR and meta packages were employed using R software (version 4.3.1) for all the analyses.

## Results

### Participants and insomnia prevalence

Our analysis utilised summary-level data from multiple large-scale GWASs. Participants included 5959 European-descent individuals from the FINRISK study, with an average age of 45.7 years (SD = 11.5), among whom 50% were male. Plasma metabolites data came from the CLSA, encompassing 8299 European-descent individuals aged between 45 to 85 years (mean age = 63 years), with males constituting 52%. Insomnia data were aggregated from three sources: FinnGen (4801 cases and 405,229 controls; mean age = 53 ± 18 years, 44% males), GERA (3,972 cases and 52,665 controls, mean age = 63 years, ranging from 19 to over 100 years, 42% males), and Pan-UKB (234 cases and 413,908 controls; mean age = 55.1 ± 7.6 years, 48% males). The combined prevalence of insomnia across these populations was approximately 1.02% (9007 cases out of 880,809 total participants).

### Gut microbiota and insomnia

**Figure 2.**
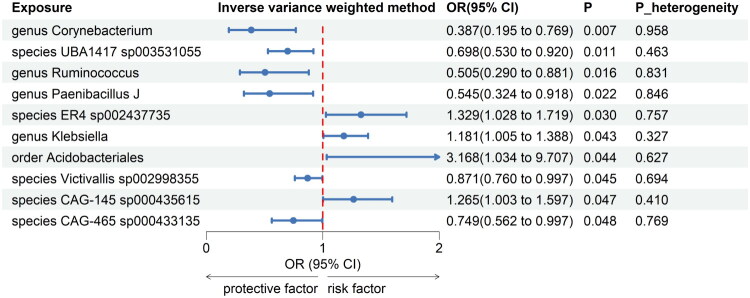
Forest plot of forward MR analysis between gut microbiota and insomnia. OR: odds ratio; CI: confident interval; P_heterogeneity: *p* value of heterogeneity for meta-analysis.

**Figure 3.**
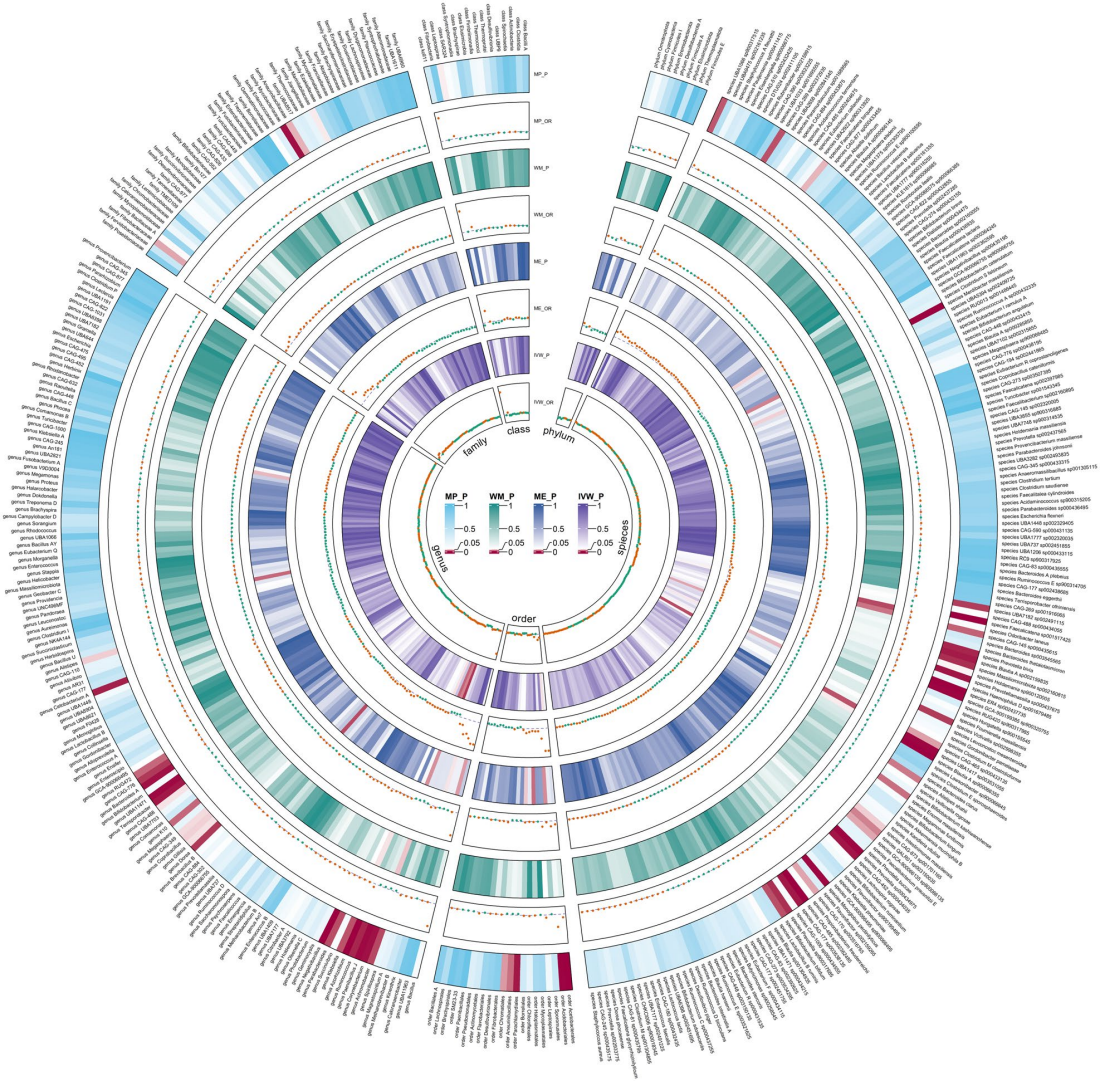
Circular heat map of meta-analysis of genetic correlation between gut microbiota and insomnia. IVW: Inverse-Variance Weighted; ME: MR-Egger; WM: Weighted median; MP: MR-PRESSO. The colour variations represented the size of the *p* value. The scatter plots reflect OR, with OR > 1 labelled red and OR < 1 labelled green.

Meta-analyses of 416 bacterial taxa were conducted (Supplementary Table S3). Finally, we identified 10 gut microbiomes suggestively associated with insomnia. The combined IVW estimates showed that genetically predicted species *CAG-145 sp000435615* (OR = 1.265, 95% CI 1.003, 1.597; *p* = 0.007) and 3 other gut microbiomes were suggestively associated with an increased risk of insomnia. Additionally, we found that genetically predicted genus *Corynebacterium* (OR = 0.387, 95% CI 0.195, 0.769; *p* = 0.007) and 5 other gut microbiomes were suggestively associated with a decreased risk of insomnia (Figure 2). All main results and sensitivity analyses were depicted in Figure 3.

Reverse MR analyses were conducted between insomnia and previously identified 10 gut microbiomes (Supplementary Table S4). The combined IVW estimates did not detect bidirectional causal relationship between insomnia and the previously identified bacteria.

Due to low heritability and small sample sizes, only 4 previously identified gut microorganisms were suitable for LDSC analysis. However, we did not detect genetic correlations between insomnia and the previously identified bacteria. Detailed information regarding all genetic correlation results is listed in Supplementary Table S5.

### Plasma metabolites and insomnia

**Figure 4.**
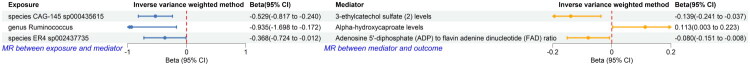
Forest plot of reverse MR analysis between identified gut microbiota and insomnia. IVs: instrumental variables; CI: confident interval; P_heterogeneity: *p* value of heterogeneity for meta-analysis; P_Q: *p* value for Cochran Q test; P_intercept: *p* value for MR-Egger intercept test; P_global: *p* value for Global test.

Supplementary Table S6 displays the results of the forward MR study, identifying 35 plasma metabolites genetically predicted to have a potential causal link to insomnia. Subsequently, 350 MR analyses (35 × 10) were conducted to examine the relationships between the identified bacterial taxa and plasma metabolites (Supplementary Table S7). A total of 3 pairs of gut microbiomes and plasma metabolites were included in mediation analyses (Figure 4). Among these, genetic predisposition to species *CAG-145 sp00043561*5 was causally linked to 3-ethylcatechol sulphate levels (Beta = −0.529 95% CI −0.817, −0.240; *p* = 3.36 × 10^−4^).

### Mediation analysis

Mediation analyses were conducted for 3 specific pairings of gut microbiomes and plasma metabolites (Supplementary Table S8). Specifically, the species *CAG-145 sp000435615* indirectly influenced insomnia through 3-ethylcatechol sulphate levels, with an ME of 0.074 (95% CI 0.006, 0.141, *p* = 0.032). The mediated proportion was 31.49%.

### Sensitivity analysis, pleiotropy, and heterogeneity

During data processing, we excluded IVW estimates that were inconsistent with the sensitivity analysis (weighted median, MR-Egger, and MR-PRESSO) or exhibited significant pleiotropy (*p* for intercept < 0.05 or *p* for global test < 0.05) to maintain the stability and validity of our findings. Most SNPs (Cochran’s Q test) and the meta-analysis results showed no or mild heterogeneity, demonstrating the robustness of our analyses.

## Discussion

Our study integrates MR and LDSC to investigate the relationship between gut microbiota, plasma metabolites, and insomnia, employing GWAS data. Forward MR analysis identified several gut microbiomes associated with insomnia. Notably, the genetic predisposition to species *CAG-145 sp000435615* was suggestively linked to an increased risk of insomnia. Conversely, the genus *Corynebacterium* was associated with a decreased risk of insomnia. Mediation analysis revealed that plasma metabolites such as 3-ethylcatechol sulphate mediate this relationship, accounting for about 31.49% of the effect on insomnia, highlighting the significant role of gut microbiota in sleep regulation and potential therapeutic avenues.

Observational studies have revealed significant associations between specific gut microbiota and insomnia. Individuals with insomnia show distinct alterations in their gut microbiota composition compared to healthy controls. An important finding is that insomnia is linked to a decrease in the richness and diversity of microorganisms, specifically a decrease in the number of anaerobes and bacteria that produce SCFA, as well as an increase in the number of potential pathobionts like *Blautia* and *Lachnospira* in patients who suffer from chronic insomnia (Li et al. 2020). Additionally, higher levels of *Bacteroides* have been correlated with poor sleep quality in individuals with depression and anxiety disorders, indicating a link between gut microbiota and sleep disorders beyond insomnia (Tanaka et al. 2023). Animal research using faecal microbiota transplantation (FMT) has shown that transferring gut microbiota from insomniacs or stressed animals to healthy ones can induce insomnia-like symptoms. Studies have demonstrated that gut bacteria such as *Lactobacillus* and *Bifidobacterium* play crucial roles in regulating sleep and stress responses (Kelly et al. 2016). Furthermore, animal models have revealed that specific microbial communities, such as those enriched with *Bacteroidetes*, can influence sleep patterns through their interaction with the host’s immune and metabolic pathways (Si et al. 2022). MR studies, which help infer causality, have shown that certain taxa of the gut microbiota are associated with the risk of insomnia. Specifically, the *Ruminococcaceae* family and the *Lachnospiraceae* genus were observed to raise the risk of insomnia, whereas the *Flavonifractor* and *Olsenella* genera seemed to mitigate this risk (Yang et al. 2023). Another Mendelian randomisation study revealed that the class *Negativicutes* and the genera *Clostridium innocuum group* and *Dorea* were linked to higher insomnia risk, whereas some taxa like *Bifidobacterium* showed potential protective roles (Li et al. 2023). Collectively, these studies underscore the critical role of gut microbiota in sleep regulation and suggest that specific microbial alterations could serve as potential biomarkers and therapeutic targets for insomnia.

The species *CAG-145 sp000435615*, belonging to the phylum *Firmicutes*, class *Clostridia*, order *Peptostrepto­coccales*, family *Anaerovoracaceae*, and genus *CAG-145*, is an uncultured species defined from metagenome-assembled reference genomes (Qin et al. 2022). Unfortunately, there isn’t readily available detailed information on this particular species in popular biological databases or literature. However, certain newly identified genera within the family *Anaerovoracaceae*, such as *Anaerohalosphaera lusitana* and *Limihaloglobus sulfuriphilus*, are characterised by their ability to utilise sulphur compounds. This utilisation may decrease sulphate levels (Pradel et al. 2020). This was in accordance with our findings, which demonstrated that the species *CAG-145 sp000435615* was associated with an increased risk of insomnia, mediated through decreased 3-ethylcatechol sulphate levels. 3-ethylcatechol sulphate is a derivative of catechol, a type of phenol, which is ethylated and then sulphated. Research suggests that metabolic byproducts from gut microbiota, such as 3-ethylcatechol sulphate, can influence brain function and behaviour through the gut-brain axis. This axis involves bidirectional communication pathways that include endocrine, immune, and neural mechanisms. A decrease in these metabolites might disturb this communication and contribute to sleep disorders (Foster and McVey Neufeld 2013). Additionally, 3-ethylcatechol sulphate might be involved in the metabolism of neurotransmitters such as dopamine, which plays a crucial role in regulating sleep-wake cycles. Catecholamines like dopamine are essential for maintaining wakefulness, and disruptions in their metabolism can affect sleep patterns. The sulphate conjugates of these neurotransmitters (like 3-ethylcatechol sulphate) are often involved in their degradation and excretion. Thus, lower levels could indicate altered dopamine metabolism, potentially leading to disturbances in sleep architecture or the ability to maintain wakefulness (Pandi-Perumal et al. 2008). There is also a theory linking oxidative stress and sleep regulation. Catechols and their metabolites have antioxidative properties. A decrease in compounds like 3-ethylcatechol sulphate could imply a reduced capacity to neutralise oxidative stress, which has been associated with poor sleep quality and insomnia (Reiter et al. 2014). The association between decreased 3-ethylcatechol sulphate levels and increased risk of insomnia aligns with current theories that emphasise the roles of metabolic imbalances, inflammatory processes, and neurobiological stress responses in the pathophysiology of insomnia. These findings suggest that targeting metabolic and inflammatory pathways might offer new therapeutic avenues for managing insomnia.

Our research offers several strengths. First, the study utilises MR analysis, a robust statistical method that helps infer causal relationships, minimising confounding factors and reverse causality. Second, the research draws from publicly available GWAS involving large sample sizes. It includes over 9000 insomnia cases and over 871,000 controls, enhancing the study’s statistical power and reliability. Third, the study explores the gut microbiota-insomnia link using forward and reverse MR analyses and also investigates plasma metabolites’ mediation role, allowing for a more nuanced understanding of the microbiota-sleep health relationship. Last, the use of meta-analyses, sensitivity analyses (e.g., MR-Egger, weighted median, MR-PRESSO), and genetic correlation testing *via* LDSC strengthen the credibility of the results. These methods detect and account for pleiotropy and heterogeneity, ensuring that the findings are stable and valid.

Several limitations of MR studies must be considered when interpreting our findings. First, the phenotypic definitions of traits such as insomnia and gut microbiota composition may not fully capture their complexity. Although large-scale GWAS data were used, phenotype data may lack precision due to self-report biases or diagnostic inconsistencies. Second, MR studies assume that genetic variants are associated with exposures measured at a single time point, overlooking time-varying factors such as fluctuations in gut microbiota or plasma metabolites. This limitation may hinder the detection of causal relationships influenced by environmental changes. Third, gene-environment interactions could impact associations between gut microbiota, plasma metabolites, and insomnia. While MR reduces confounding, it cannot fully account for environmental influences such as diet, stress, or medication use, which may introduce residual confounding. Fourth, measurement errors in exposures or outcomes may introduce bias. Despite using large-scale GWAS data, variations in analytical techniques and sample handling could affect the accuracy of gut microbiota and plasma metabolite measurements, impacting result robustness. Reverse causation is also a concern, as insomnia could influence gut microbiota and metabolite levels, potentially confounding observed associations. Fifth, LD may affect results. While we selected SNPs with low LD to minimise this issue, residual effects could still bias estimates. Additionally, pleiotropy may influence findings, as some genetic variants may affect insomnia through mechanisms beyond those considered. Although sensitivity analyses were conducted, unmeasured pleiotropic effects cannot be entirely ruled out. Lastly, the observed insomnia prevalence of ∼1% in our GWAS sample is much lower than epidemiological estimates of 10%–30%, possibly due to differences in diagnostic criteria, data collection methods, or reliance on clinical diagnoses that may overlook subclinical cases. Population-specific factors such as age, culture, healthcare access, and reporting biases may also contribute. These limitations highlight the need for careful interpretation and further validation through independent studies. Future research using standardised diagnostic tools and broader inclusion criteria may enhance generalisability.

This study underscores the significant impact of gut microbiota and plasma metabolites on insomnia. Moreover, plasma metabolites, notably 3-ethylcatechol sulphate, were found to mediate the relationship between species *CAG-145 sp00043561*5 and insomnia, explaining up to 31.49% of the effect. These findings highlight the intricate interplay between gut microbiota, plasma metabolites, and sleeping condition, providing valuable insights into potential therapeutic targets. Future research should aim to elucidate the precise mechanisms underlying these interactions. Additionally, exploring therapeutic interventions that modify the gut microbiota could help prevent and treat insomnia.

## Supplementary Material

STROBE MR checklis.docx

Supplementary tables.xlsx

## Data Availability

The datasets presented in this study can be found in online repositories. The names of the repository/repositories and accession sites can be found in the article. The data that support the findings of this study are openly available in Figshare at https://doi.org/10.6084/m9.figshare.27061420.v1. If more information is needed, the corresponding author can be contacted.
